# Synergistic effects of ultrasound and stirring on crystallization behavior of coconut oil: Physicochemical properties, crystallinity and potential mechanism

**DOI:** 10.1016/j.fochx.2026.104068

**Published:** 2026-06-09

**Authors:** Huiting Yang, Yuting Fu, Siyuan Xu, Jingya Xue, Jun Cao

**Affiliations:** Key Laboratory of Food Nutrition and Functional Food of Hainan Province, School of Food Science and Engineering, Hainan University, Haikou 570228, China

**Keywords:** Coconut oil, Ultrasound and stirring, Crystallization behavior, Polymorphism, Microstructure, Textural property

## Abstract

Precise control of coconut oil crystallization is essential for tailoring its structural and functional attributes. However, chemical modification often encounters constraints, and mechanisms of physical modulation, such as ultrasound and stirring, remain to be fully explored. In this work, the independent and combined effects of ultrasound and mechanical stirring on the crystallization of virgin coconut oil (VCO) and refined, bleached, deodorized coconut oil (RBD) were investigated. All treatments accelerated nucleation and crystal growth, with the combined field showing the strongest effect. X-ray diffraction confirmed enhanced formation of β' crystals, which dominated polymorphic transitions. This led to hierarchical structural refinement: thinner nanocrystallites and finer, more uniform spherical aggregates. The optimized crystal network exhibited higher fractal dimension, reduced melting temperature, and increased hardness, particularly under synergistic treatment. These results demonstrate that integrated ultrasound and stirring offer an effective physical strategy to direct crystallization, providing insights into designing structured lipids with tunable properties.

## Highlights

• Comparison of ultrasound, stirring, and synergy reveals distinct crystal effects.
• Ultrasound–stirring synergy boosts early-stage nucleation and crystal growth.
• Synergy favors β' polymorph and reduces nanoscale crystallite thickness.
• Synergy forms finer, uniform, and compact crystal networks.
• Synergistic treatment significantly enhances hardness of coconut oil fats.

## 1 Introduction

Coconut oil, characterized by a high proportion of lauric acid and medium-chain triacylglycerols (TAGs), has been increasingly regarded as a healthy lipid and is widely used in the development of structured fat systems, including cocoa butter substitutes, margarine and frozen desserts (Liu et al., 2018). Coconut oil is generally classified into virgin coconut oil (VCO) and refined, bleached and deodorized coconut oil (RBD). Although the two come from the same raw materials, the differences in extraction and purification processes lead to significant differences in their composition and physicochemical properties (Chang et al., 2020). Structurally, coconut oil is composed of a variety of fatty acids and TAGs. Its high saturation leads to a wide range of melting points and complex crystallization properties (Singh & Hebbar, 2024). The crystallization process of fat involves two essentially interrelated stages: nucleation and subsequent crystal growth. In the actual system, these stages occur at the same time, leading to polymorphic transformation and changes in crystal size and network structure. These microstructural characteristics are crucial to the design of fat-based products, because they have a significant impact on texture, sensory performance, stability and overall appearance (Chen et al., 2025).

In the current research, the crystallization regulation of coconut oil mainly depends on chemical methods. For example, Mahisanunt et al. (2019) proved that exogenous high-melting-point triglycerides such as glyceryl tripalmitate and glyceryl tristearin could effectively increase the crystallization temperature of coconut oil or directly induce the formation of stable β' polymorph. Similarly, Chai et al., 2018a, Chai et al., 2018b reported that endogenous non-triacylglycerol molecules, including free fatty acids and diacylglycerols, could alter nucleation and crystal growth kinetics via selective adsorption onto lattice sites. Additional studies further indicated that external surfactants such as sorbitan esters, monoglycerides, and sucrose esters modify crystallization behavior through specific lattice interactions (Chaleepa et al., 2010b; Maruyama et al., 2014; Sonwai et al., 2016). However, traditional chemical methods inevitably change the intrinsic structure of the lipid system by adding additives or removing endogenous components. In addition, their limited controllability and potential environmental impact have prompted people to have a growing interest in physical methods that regulate crystallization through external fields rather than chemical changes (Misra et al., 2013).

Research showed that low-frequency ultrasonic processing can significantly enhance nucleation due to cavitation; however, problems about frequency compatibility and temperature control still limit its industrial applications (Chen et al., 2013; Silva et al., 2017). In order to alleviate these limitations, recent research has combined ultrasonic processing with temperature gradient field, thus establishing a physical regulation strategy, which is more effective than simple chemical regulation (Devos et al., 2025; Xiang et al., 2024). Recently, the space utilization of high-intensity ultrasound (HIU) in scraper heat exchangers has attracted more and more attention. Evidence shows that the application of HIU in the early stage of crystallization, especially under low temperature and limited stirring conditions, can significantly improve the physical quality of palm-based fat (Da Silva et al., 2021). Meanwhile, relevant studies have also utilized ultrasound in the later stage to further refine the crystal network of soybean oil (Da Silva et al., 2020). Although physical field-assisted technology has been proven to be effective in regulating fat crystallization, there are still few comprehensive studies specifically targeting coconut oil, which possesses unique crystallization properties. In this study, an innovative non-chemical method utilizing a synergistic dual-physical field was developed, combining ultrasound and mechanical stirring to control the crystallization of coconut oil. Distinct from previous studies focused on single-field applications, this approach leverages the synergistic coupling between cavitation and shear-induced flow. This allows for precise, multi-stage regulation of the crystallization process without destroying the inherent molecular structure, thus providing a sustainable way for the directional design of structured coconut oil-based lipids.

The focus of this study was to establish a precision regulation strategy for coconut oil crystallization. Therefore, this study systematically investigated the effects of ultrasound, mechanical stirring and their synergistic application on the melting and crystallization of two coconut oils (VCO and RBD) to clarify their roles in regulating crystallization behavior and physicochemical properties. The research process integrated multi-scale structural characterization, including lipid composition analysis, crystallization process kinetics, polymorphism, microstructure observation, thermal property evaluation and textural analysis. This study aimed to elucidate how physical modulation affects the mechanisms of nucleation and crystal growth, and to reveal the intrinsic structure-functional relationship of coconut oil crystallization. In addition, these findings provide a theoretical basis for non-chemical modification strategies and support the rational design of customized functional lipid products.

## 2 Materials and methods

### 2.1 Materials

VCO was obtained from Wenchang Yefu Industry and Trade Co., Ltd. (Wenchang, China). RBD was supplied by Hebei Tianyi Edible Oil Co., Ltd. (Hebei, China). Fatty acid methyl ester (FAME) standard mixtures (GLC-463) were purchased from NuChek-Prep (Elysian, MN, USA). Trilaurin (standard grade), isopropanol (chromatographic grade), n-hexane (chromatographic grade), acetonitrile (chromatographic grade), and other analytical-grade chemicals and reagents were purchased from Sigma-Aldrich (St. Louis, MO, USA).

### 2.2 Lipid composition analysis

#### 2.2.1 Fatty acid composition

Fatty acid composition was determined using a GC system (Agilent 7890A, Agilent Technologies, USA) equipped with a flame ionization detector and a CP-Sil 88 capillary column (100 m × 0.25 mm inner diameter, 0.20 μm film thickness). Fatty acids were first converted into their methyl esters according to the method reported by Cui et al. (2024). The temperature program was as follows: initial temperature at 45 °C for 4 min; ramped from 45 °C to 175 °C at 13 °C/min and held for 27 min; then ramped to 215 °C at 4 °C/min and held for 35 min. The injection volume was 1 μL, and hydrogen served as the carrier gas. The detector was supplied with hydrogen and air, while nitrogen was used as the make-up gas. FAMEs were identified by comparing retention times with those of authentic mixed standards, and quantification was performed using the area normalization method.

#### 2.2.2 Triacylglycerol composition

The composition of TAGs was analyzed using an Agilent 1260 HPLC system (Agilent Technologies, USA) equipped with an evaporative light scattering detector (ELSD) and a ZORBAX Eclipse Plus C18 column (250 × 4.6 mm, 5 μm). The analysis method was based on Liu et al. (2023). The method described had been modified to suit the characteristics of the sample. The mobile phase consisted of acetonitrile (solvent A) and isopropanol (solvent B). The elution program was as follows: from 0 to 30 min, 30–40% A; from 30 to 70 min, 40–45% A; from 70 to 100 min, 45% A; from 100 to 105 min, back to 30% A. The column temperature was maintained at 30 °C, and the ELSD drift tube at 55 °C. The flow rate was 0.8 mL/min, and the injection volume was 10 μL. TAG species were identified based on their equivalent carbon number (ECN), calculated using the formula ECN = CN − 2 × DB, where CN is the total carbon number and DB is the number of double bonds (Da Silva et al., 2020). The elution order was predicted by ECN values and confirmed with literature data. Quantification was performed using the area normalization method.

### 2.3 Melt crystallization process

The experimental setup in this study was based on Chaleepa et al. (2010a) and consists of a temperature-control system, a cold finger (3 cm in diameter), a thermostatic magnetic-stirring water bath, an ultrasonic generator, a digital thermometer, and a 100 mL glass beaker. All melt crystallization experiments were performed using this laboratory-scale temperature-gradient field apparatus. Briefly, 50 g of coconut oil was placed in a 100 mL glass beaker, heated to 60 °C, and allowed to equilibrate for 20 min. The oil was transferred into the crystallization chamber and maintained at 32 °C in a molten state throughout the experiment by means of a thermostatic water bath. Crystallization was induced by a cold finger (3 cm in diameter) immersed in the oil, the surface temperature of which was programmed to decrease from 32 °C to 17 °C at a controlled cooling rate of 8 °C/h using a programmable temperature controller connected to the cold finger circulation system (Wu et al., 2016). The system was maintained under isothermal conditions at this temperature for 8 h, and the mass of the crystal growth layer was recorded every hour to monitor the crystal growth rate and crystallization yield.

Six different experimental groups were designed to systematically evaluate the effects of ultrasound, mechanical stirring and their combined applications on crystallization behavior. In the ultrasonic pretreatment group (UP), samples were treated with ultrasound for 240 s in the molten state (32 °C) using an ultrasonic generator (KQ5200E, Kunshan Ultrasonic Instruments Co., Ltd., China) operating at 40 kHz with a power input of 200 W, corresponding to a nominal power density of approximately 3.64 W/mL (Silva et al., 2017; Wu et al., 2016). For the ultrasonic treatment group (U), ultrasound was applied at the critical crystallization point (21 °C) using the same parameters. The critical crystallization point was defined as the temperature at which the first visible crystal nuclei appeared under the specified cooling conditions, and the application of ultrasound at this stage has been shown to be more effective (Suzuki et al., 2010). During sonication, the sample temperature was monitored using a digital thermometer. The mechanical stirring group (M) was stirred continuously at 200 rpm throughout the crystallization process (Chaleepa et al., 2010b; Youngs et al., 2021). In the ultrasonic pretreatment combined with mechanical stirring group (UP-M), samples were subjected to ultrasound first, followed by stirring at 200 rpm during crystallization. In the ultrasonic treatment at the critical crystallization point combined with mechanical stirring group (U-M), continuous mechanical stirring at 200 rpm was maintained throughout the crystallization, and ultrasound was applied separately at the critical crystallization point. The control group (CK) was crystallized without any external treatment. Each experiment was performed in triplicate.

### 2.4 Crystallization yield

The crystallization yield was calculated via the following Eq. (1):

(1) $$\text{Crystallization yield}=\frac{M_{1}}{M_{0}}\times 100\;\left(\%\right)$$

where *M*_*1*_ is the mass of the solid fraction crystallized on the cold finger's surface (g) and *M*_*0*_ is the mass of the melt (g).

### 2.5 Crystal growth rate

The crystal growth rate was calculated using the following Eq. (2) (Chaleepa, 2010a):

(2) $$R_{G}=\frac{{\mathrm{dM}}_{C}}{\mathrm{Adt}}\;\left[\mathrm{kg}\;M^{-2}\;s^{-1}\right]$$

where *R*_*G*_ is the crystal growth rate (assumed as a constant crystal growth rate), *M*_*C*_ is the mass of crystals formed on the surface of the cold finger (kg), *A* is the surface area covered by the crystals (m^2^), calculated as *A* *= 2πrl + πr*^*2*^, with *r* being the radius of the rod (m) and *l* is the height of the crystal layer formed on the rod surface (m). *t* is the crystallization time (s).

### 2.6 Crystal polymorphism and crystallite thickness analysis

After isothermal crystallization at the designated temperature for 48 h, the crystalline forms of the samples were characterized using an X-ray diffractometer (Smart Lab SE, Rigaku, Japan) equipped with a Cu-Kα radiation source. Small-angle and wide-angle diffraction patterns were collected in the 2θ range of 1°–10° and 10°–30°, respectively. The measurements were performed at 40 kV and 40 mA with a scan rate of 10°/min, step size of 0.02°, and sampling interval of 0.02 s. The obtained X-ray diffraction (XRD) data were analyzed using MDI Jade software (version 9, Materials Data, Livermore, CA, USA) to assess phase composition and crystalline characteristics.

The relative content of β' polymorphs was estimated by the intensity of the characteristic peaks located at 4.20 Å and 3.80 Å, while the α and β forms were identified at 4.15 Å and 4.60 Å, respectively. The percentage of β' crystals was calculated using a peak area normalization method based on the following Eq. (3) (Zhou et al., 2024):

(3) $$\beta^{\prime }\mathrm{content}=\frac{A_{\beta \prime }}{A_{\alpha }+A_{\beta \prime }+A_{\beta }}\times 100\;\left(\%\right)$$

where *A*_α_, *A*_β′_, and *A*_β_ represent the integrated peak areas corresponding to *α* (4.15 Å), *β'* (4.20 and 3.80 Å), and *β* (4.60 Å) polymorphs, respectively.

The crystallite thickness (*ξ*) is calculated according to the Scherrer Eq. (4):

(4) $$\xi =\frac{\mathrm{k\lambda}}{\mathrm{FWHMcos\theta}}$$

where *ξ* is the crystallite thickness, *k* is the shape factor (taken as 0.9 for particles with unknown morphology), λ is the wavelength of the X-ray with the value of 1.54 Å for copper, *FWHM* is the full width at half maximum of the diffraction peak (in radians), and *θ* is the Bragg angle.

### 2.7 Crystal morphology

The microstructure of coconut oil crystals was observed using a polarized light microscope (PLM; MJ31, Guangzhou Mingmei Optoelectronics Technology Co., Ltd., China) equipped with an MDX10 digital microscope camera. A 10 μL aliquot of each sample, obtained under different crystallization conditions, was placed onto a cleaned and pre-treated glass slide. After sample preparation, the slide was transferred to a PLM equipped with a temperature-controlled stage (PE40-150-A, Wentian Precision Instrument Technology Co., Ltd., China) and held at a constant crystallization temperature of 17 °C. Once the temperature stabilized, crystal morphology was examined at 100× magnification and images were captured. The fractal dimension (*D*_b_) of the crystal network was calculated using the box-counting method in *Image*J software. In addition, the average crystal size (D) was determined by measuring fifteen separate crystals in each image using the MShot Image Analysis System (version 1.2.3) (Mahisanunt et al., 2019). All experiments were repeated three times, and representative images were selected for display.

### 2.8 Thermal properties

The thermal properties of the samples were analyzed using a differential scanning calorimeter (DISCOVERY DSC250, TA Instruments, USA). Weigh about 10 mg of each sample in a standard aluminum crucible (type 70) and seal it, with an empty crucible as a reference. The samples were first held at 60 °C for 10 min to ensure complete melting and eliminate any prior crystalline memory. Subsequently, the samples were cooled to −30 °C at a rate of 10 °C/min to record the crystallization thermogram. After holding at −30 °C for 5 min, the temperature was raised to 60 °C at the same heating rate to obtain the melting thermogram.

### 2.9 Texture profile analysis

The texture properties of the samples were evaluated using a TA.XT Plus texture analyzer (Stable Micro Systems, Godalming, UK). Measurements were carried out using a cylindrical probe (P/0.5) through a double compression test. Prior to the test, the probe approached the sample at a speed of 4 mm/s, followed by a compression phase at 2 mm/s, and retraction also at 2 mm/s. A trigger force of 5.0 g was applied, and the penetration depth was set to 5 mm.

### 2.10 Statistical analysis

Data are presented as the mean ± standard deviation from three independent replicates. Statistical analyses were conducted using IBM SPSS Statistics 27. Two-way ANOVA was performed to evaluate the effects of oil type, treatment, and their interactions. Significant differences among groups were assessed by Tukey's post hoc test at *p* < 0.05 and are indicated by superscript letters. Figures were prepared using Origin 2025, and XRD patterns were analyzed with MDI JADE 9.

## 3 Results and discussion

### 3.1 Fatty acid and TAG compositions of VCO and RBD

**Fig. 1:**
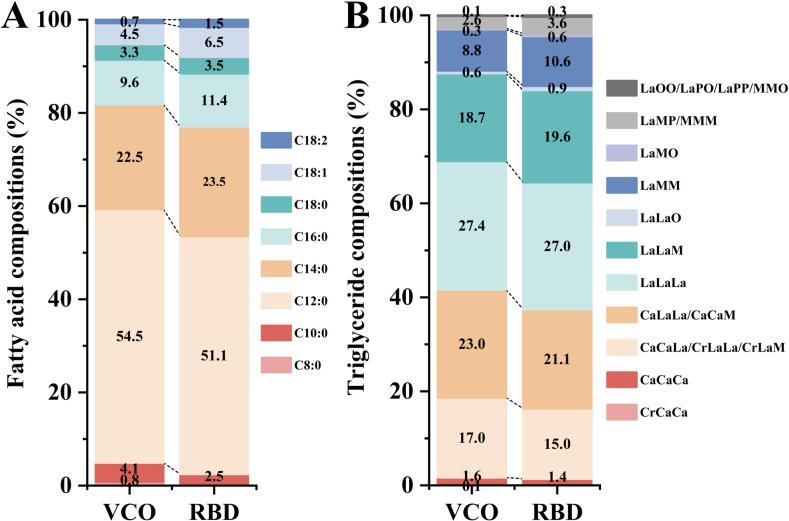
Fatty acid and triglyceride compositions of virgin coconut oil (VCO) and refined, bleached and deodorized coconut oil (RBD). C8:0 / Cr, Caprylic; C10:0 / Ca, Capric; C12:0 / La, Lauric; C14:0 / M, Myristic; C16:0 / P, Palmitic; C18:0 / St, Stearic; C18:1 / O, Oleic; C18:2 / L, Linoleic.

The physicochemical properties of oils and fats are largely dictated by their composition of fatty acids and TAGs. Differences in lipid composition, molecular structure and intermolecular interactions together determine their crystallization behavior and the resulting macroscopic characteristics. Fatty acid composition analysis showed that both oils were mainly saturated fatty acids (SFA), accounting for more than 90% of the total content (Fig. 1A). The content of lauric acid (C12:0) was the highest, accounting for 54.5% of VCO and 51.1% of RBD. High levels of myristic acid (C14:0), palmitic acid (C16:0) and stearic acid (C18:0) were also detected. Although saturated fatty acids were dominant, both oils contained a small amount of detectable unsaturated fatty acids, mainly oleic acid (C18:1) and linoleic acid (C18:2). The content of these unsaturated ingredients in RBD coconut oil was higher than that of VCO. Structurally, the *cis*-double bonds in unsaturated fatty acids create steric impediments that disrupt lateral acyl chain packing, which is likely to delay the induction time of crystallization and facilitate lattice defects during initial nucleation (Yang et al., 2024).

The TAG profile showed that both VCO and RBD coconut oils are complex mixtures predominantly consisting of medium-chain TAGs (Fig. 1B). Characteristic species included LaLaLa, CaLaLa/CaCaM, LaLaM and CaCaLa/CrLaLa/CrLaM, which were typical of coconut oil. While VCO and RBD shared a similar TAG profile, quantitative analysis revealed that VCO contained slightly higher levels of short-chain and medium-chain TAGs, while the proportion of unsaturated fatty acids in RBD was relatively high. Notably, crystallization is influenced not only by major TAG species but also by minor components. VCO, as a minimally processed oil, typically retains higher levels of monoacylglycerols and phospholipids, which can act as impurities that interfere with the crystal growth front. Conversely, the refining process of RBD eliminates these inhibitory components, potentially lowering the energy barrier for nucleation (Gordon & Rahman, 1991). Under the same crystallization conditions, the different crystallization behavior, thermal properties and texture characteristics of VCO and RBD are likely to be affected by these subtle variations in lipid composition.

### 3.2 Effects of different crystallization conditions on crystallization yield and crystal growth rate

**Fig. 2:**
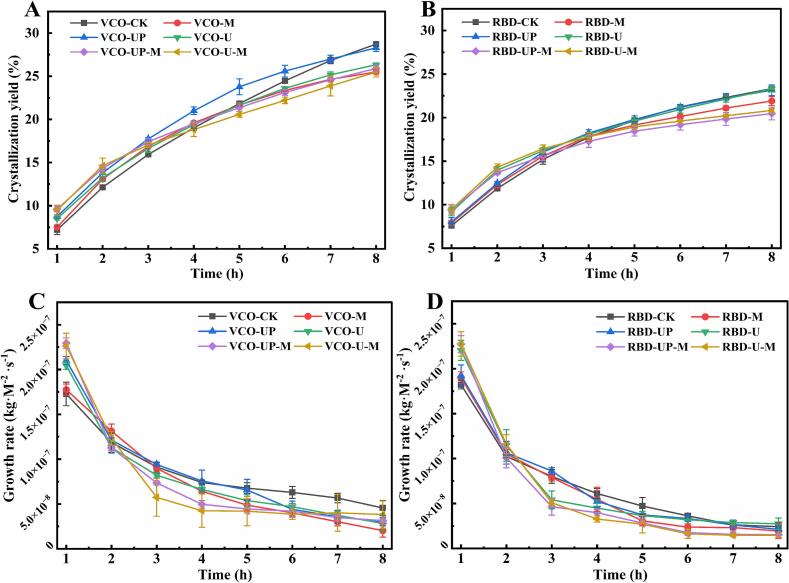
Crystallization yield (A, B) and crystal growth rate (C, D) of VCO and RBD under different crystallization treatments. VCO: virgin coconut oil; RBD: refined, bleached and deodorized coconut oil; CK: control; M: mechanical treatment; UP: ultrasonic pretreatment; U: ultrasonic treatment at a critical crystallization point; UP-M: ultrasonic pretreatment combined with mechanical treatment; U-M: ultrasonic treatment at a critical crystallization point combined with mechanical treatment.

As shown in Fig. 2, both VCO and RBD exhibited a gradual increase in crystallization efficiency with prolonged crystallization time, followed by a stabilization at the plateau stage. The earlier reach of this stable state in RBD may be related to the refining process, which can remove the minor components that hinder crystallization. The analysis of the crystal growth rate showed that the *R*_*G*_ values of VCO and RBD generally decreased over time, maintaining a critical range of 10^−8^ to 10^−7^ kg·M^−2^ ·s^−1^. This range represents a key window for process optimization, which can realize controlled crystalline dynamics, selectively separate components at the melting point, promote the formation of the required stable polymorph, and reduce entrapment of liquid oil (Sato et al., 2013).

At the early stage, all physical treatment groups exhibited significantly higher crystallization efficiency and *R*_*G*_ compared with the control, with the effect being most pronounced under synergistic treatments. This acceleration is consistent with the cavitation-induced nucleation theory, where ultrasound provides additional nuclei, a mechanism previously discussed in lipid crystallization studies (Silva et al., 2017). However, in the later stage, treatments involving mechanical stirring showed markedly lower values for both parameters compared with non-stirred groups. This may be attributed to the fact that, during the initial phase, stirring mainly enhanced nucleation, thereby increasing the number of nuclei and initial growth rates. In contrast, the shear force generated by continuous stirring in the later stage may disturb the crystal growth environment and disrupt the formed crystal network, thus reducing the spatial packing efficiency and growth rate. Indeed, excessive mechanical input can induce uncontrolled secondary nucleation and significant heat generation, which disrupts the ordered assembly of triacylglycerols and hinders the formation of a well-consolidated crystalline network (Singh & Hebbar, 2024). Therefore, the advantages of ultrasound-stirring synergy are mainly reflected in the nucleation stage (Zhang et al., 2024), and its overall effect requires further optimization through precise adjustment of process parameters.

### 3.3 Effects of different crystallization conditions on the crystal polymorphism

**Fig. 3:**
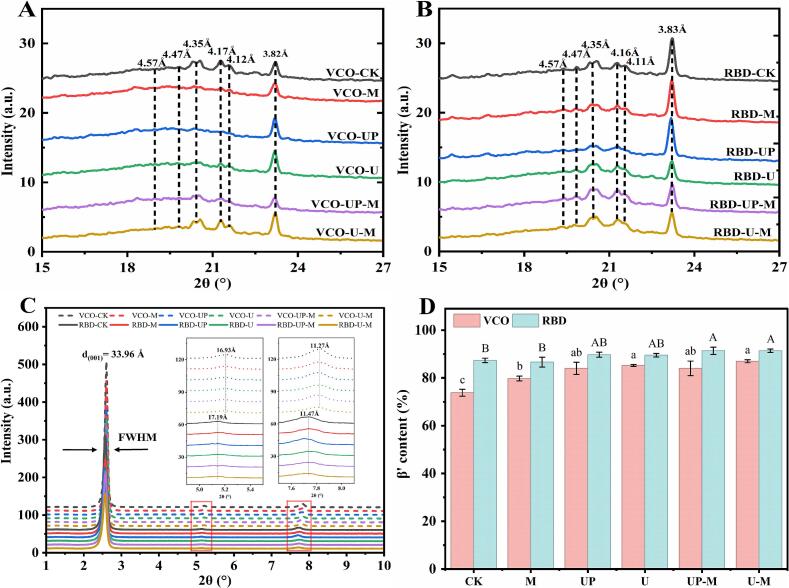
XRD patterns and polymorph analysis of VCO and RBD under different crystallization treatments including WAXD patterns for short spacings of VCO (A) and RBD (B), SAXD patterns for long spacings of both VCO and RBD (C), and changes in β' polymorph content (D). Different lowercase letters indicate significant differences for VCO, and different uppercase letters indicate significant differences for RBD (*p* < 0.05). FWHM: full width at half maximum.

X-ray diffraction (XRD) was employed to characterize the polymorphic behavior and nanostructure of the crystallized coconut oils. Short-spacing signals were used to identify specific polymorphs of TAGs, including α, β' and β modifications. XRD analysis showed that the α form appeared at 4.12 Å, the β' polymorph appeared at 4.20 and 3.80 Å, and the β polymorph appeared at 4.60 Å, corresponding to loose, medium and densely stacked crystal structures respectively. Short-spacing XRD scans (Fig. 3A and B) showed diffraction peaks at 3.82, 4.12, 4.17, 4.35, 4.47 and 4.58 Å, corresponding to α, β and β' polymorphs (Fu et al., 2025). This polymorphism may stem from changes in the wide distribution of fatty acid chains, structural asymmetry and the degree of fatty acid unsaturation in TAGs (Yang et al., 2024). The obvious XRD peaks unique to the β' polymorph at 3.80 Å and 4.20 Å, and the additional diffraction corresponding to β'-2 at 4.35 Å and 4.47 Å, indicated that the β' polymorph dominated the coconut oil crystals, with intensities significantly higher than those of the α phase and β phase. The long spacing XRD spectrum showed a characteristic peak at 33.96 Å (d_001_), indicating that the β' polymorph possessed a double-chain length (2 L) structure (Chai et al., 2018a; Sonwai et al., 2016). The additional low-intensity diffraction peaks (Fig. 3C) confirmed the existence of second-order (d_002_ at 16.93 and 17.19 Å) and third-order (d_003_ at 11.27 and 11.47 Å) diffraction peaks, which further supported the long-range longitudinal order of the crystal lattice, despite the observation of some incomplete structural arrangements (Chai et al., 2018a, Chai et al., 2018b).

The polymorph quantification (Fig. 3D) obtained from the wide-angle X-ray diffraction (WAXD) characteristic peak (Eq. 3) showed that the synergistic treatment (U-M) significantly increased the content of β' polymorph compared to the control (CK). This suggests that the cavitation and shear force from the combined physical field could facilitate the formation of the β' polymorph by guiding the crystallization toward its sub-steady-state development (Nguyen et al., 2020). The crystallite thickness determined by the Scherrer equation decreased significantly (*p* < 0.05) in all treatment groups compared with the control (Table 1). The crystallite thickness of VCO-CK and RBD-CK was 842.8 Å and 687.5 Å, respectively. The reduction under synergistic treatment (U-M) was the most significant (*p* < 0.05), reducing the crystallite thickness of VCO and RBD to 710.7 Å and 588.2 Å respectively. The reduction of nanometer-level grain thickness could be attributed to the combined effect of ultrasonic cavitation and shear-induced fragmentation, which potentially inhibited the longitudinal stacking of triglyceride molecules and promoted a more uniform layered structure (Devos et al., 2025).

**Table 1:** Crystallite thickness of VCO and RBD under different crystallization treatments.

Groups	VCO (Å)	RBD (Å)
CK	842.8 ± 11.8^aA^	687.5 ± 9.1^aB^
M	828.7 ± 19.9^abcA^	668.6 ± 5.5^aB^
UP	836.1 ± 16.1^abA^	641.1 ± 2.4^bB^
U	792.2 ± 8.8^cA^	634.0 ± 0.7^bB^
UP-M	798.8 ± 6.0^bcA^	631.0 ± 9.6^bB^
U-M	710.7 ± 20.6^dA^	588.2 ± 8.7^cB^
Oil type (O)	**	
Treatment (T)	**	
Interaction (O × T)	**	

Results are presented as means ± standard deviation (*n* = 3). Different lowercase letters indicate significant differences among treatments and different capital letters indicate significant differences between oil types (*p* < 0.05). *p < 0.05, **p < 0.01. VCO: virgin coconut oil. RBD: refined, bleached and deodorized coconut oil. CK: control. M: mechanical treatment. UP: ultrasonic pretreatment. U: ultrasonic treatment at a critical crystallization point. UP-M: ultrasonic pretreatment combined with mechanical treatment. U-M: ultrasonic treatment at a critical crystallization point combined with mechanical treatment.

### 3.4 Effects of different crystallization conditions on the crystal morphology

**Fig. 4:**
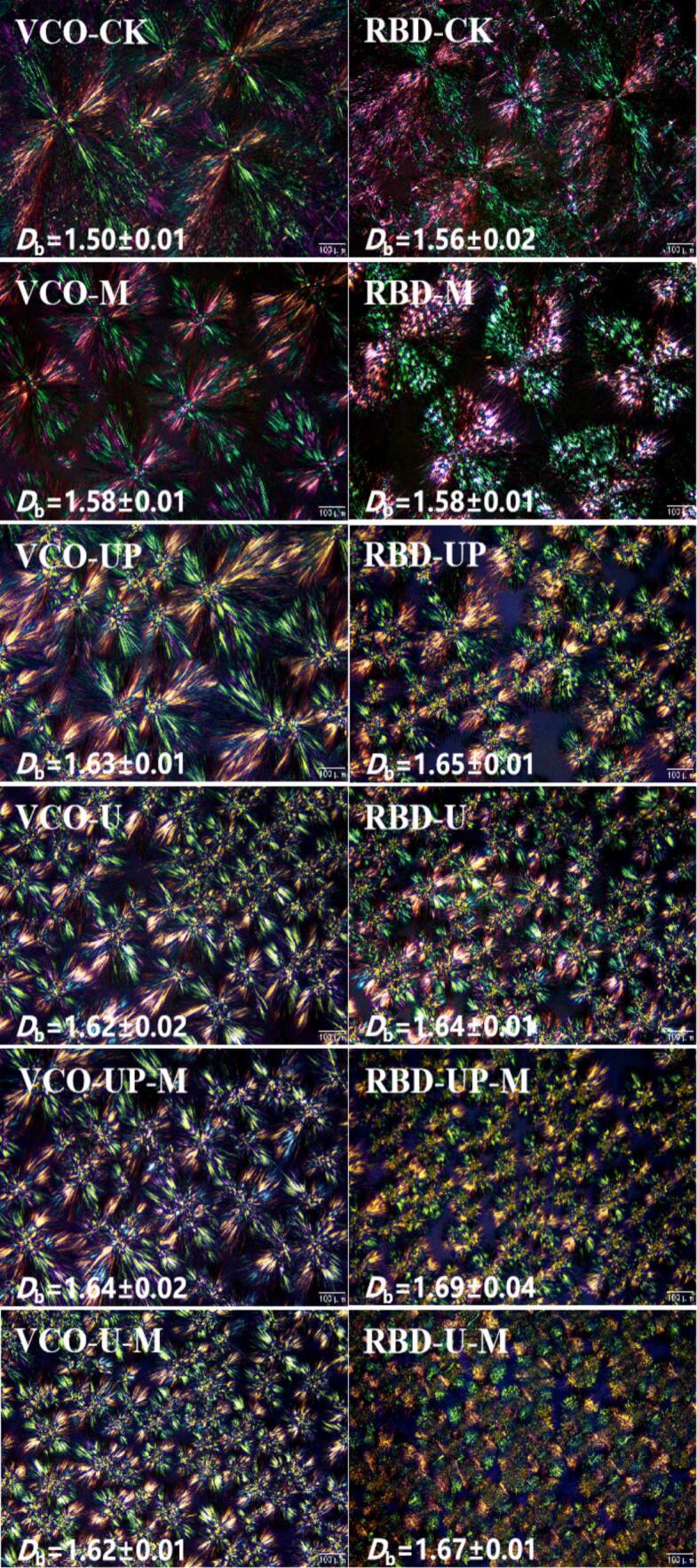
Polarized light micrographs of VCO and RBD samples under different crystallization treatments. The scale bar represents 100 μm. *D*_b_: fractal dimension.

**Fig. 5:**
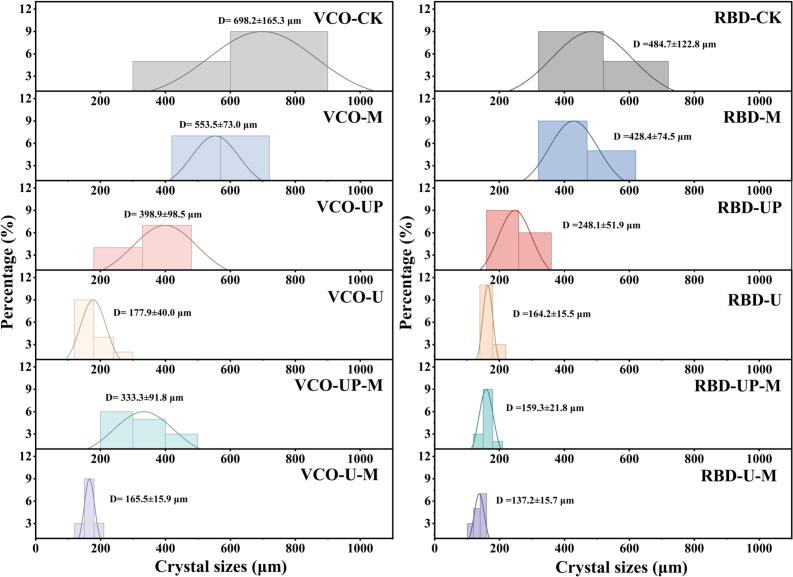
Crystal size distribution of VCO and RBD under different crystallization treatments. D: the average crystal size**.**

Polarized light micrographs showed that under the same crystallization conditions, the coconut oil crystal aggregates exhibited obvious morphological changes. Compared with the control group, the crystal density increased and the average crystal size decreased (Fig. 4). Coconut oil crystals showed a characteristic Maltese cross pattern, with radial needle-shaped crystals extended outward from the nucleus. Both the CK group and the M group showed loosely packed spherical crystals composed of macroscopic needle-shaped crystals. The irregular packing of these large crystals trapped a large amount of liquid oil in the crystal network, which hindered effective phase separation (Chaleepa et al., 2010a). The crystal size distribution curve showed that both VCO and RBD showed a similar decreasing trend, and the treated samples had smaller crystal sizes and narrower distribution than the control group. This effect was more pronounced in RBD (Fig. 5). There was no obvious morphological difference between the mechanical stirring group and the control, but its crystal size decreased, which may be due to crystal fracture and further growth inhibition.

When ultrasound and stirring were applied at the same time in the critical crystallization temperature range, the combined effects of shear force and cavitation inhibited the orderly growth of large spherical crystals and promoted the formation of finer crystals. The quantitative analysis of crystal morphology revealed that this synergistic treatment significantly increased the nucleation density, as evidenced by a substantial reduction in the average crystal size from 698.2 μm of VCO-CK and 484.7 μm of RBD-CK to 165.5 μm of VCO-U-M and 137.2 μm of RBD-U-M respectively (*p* < 0.05). These smaller crystals exhibited irregular shapes, thus reducing the size of aggregates and forming a denser and finer crystal network, resulting in a more uniform crystal distribution (Nguyen et al., 2020).

The higher fractal dimension (*D*_b_) indicated that the crystal arrangement had a higher structural order, indicating that the nucleation and growth process occurred in a more organized way (Chai et al., 2019). The *D*_b_ values of RBD were always higher than those of VCO, indicating that the removal of impurities in the refining process promoted the formation of a more orderly crystal network (Mohammed et al., 2021). In addition, the *D*_b_ value of the control group was the lowest, indicating that its crystal network was relatively rough and loose. The crystal size was large and the distribution was more uneven, which led to the formation of clusters. This situation was conducive to the formation of large but loose microstructures, thus reducing the efficiency of spatial packing. The *D*_b_ values of the treated groups increased significantly (*p* < 0.05), especially in the synergistic treatment groups, RBD-UP-M and RBD-U-M reached 1.69 and 1.67 respectively. This marked increase in *D*_b_ further quantitatively confirmed the formation of a higher nucleation density and a more compact 3D crystal network. Ultrasound and stirring inhibited excessive crystal growth through shear force and cavitation effects, while promoting orderly crystallization at the same time. As a result, smaller and more densely distributed crystals were generated, giving rise to a compact network with enhanced tightness and mechanical strength (Mao et al., 2024).

### 3.5 Effects of different crystallization conditions on thermal properties

**Fig. 6:**
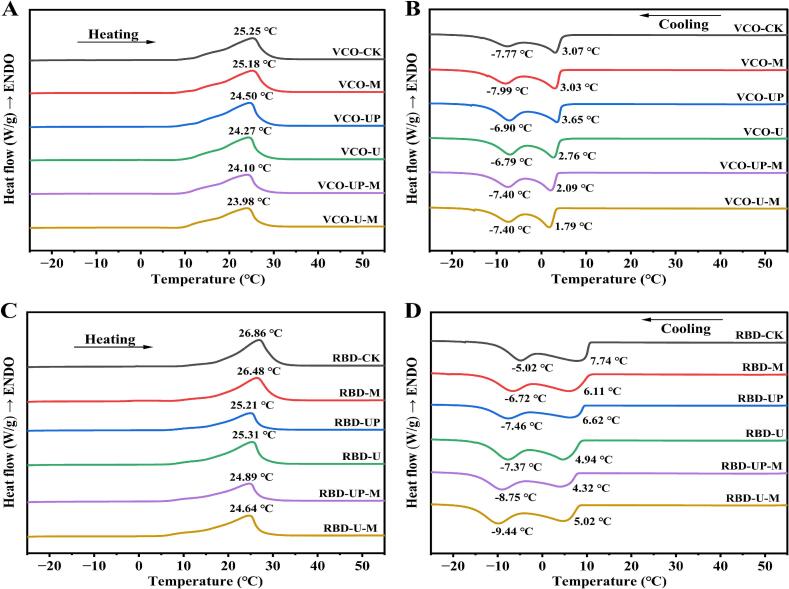
DSC melting (left side) and crystallization (right side) thermograms of VCO (A, B) and RBD (C, D) under different crystallization treatments. → ENDO: endothermic direction.

The polymorphic transitions of triacylglycerols were evaluated using differential scanning calorimetry (DSC). Fig. 6 illustrates the melting and crystallization profiles of coconut oil samples subjected to different physical treatments. As shown in the crystallization thermograms (Fig. 6B and D), two distinct exothermic peaks were observed during cooling. The first peak, appearing at relatively higher temperatures, was primarily attributed to the phase transition of medium-chain and long-chain saturated triacylglycerols enriched in lauric acid (C12:0), such as LaLaM, LaMM, LaMP, and LaLaLa. The second peak, observed below 0 °C, corresponded to the crystallization of medium-chain and short-chain as well as unsaturated TAG species, including LaLaLa, CLaLa, LaLaM, and CCLa. The melting thermograms (Fig. 6A and C) further revealed a broad melting range, where the presence of a shoulder in the initial endothermic region indicates stepwise melting behavior, which can be ascribed to variations in the chain-length distribution of lipid molecules (Arzeni & Pilosof, 2025). During cooling from 50 °C to −20 °C, coconut oil initially crystallized into the α polymorph, followed by transformation into the more compact β' and β forms. Notably, the crystallization peaks of RBD appeared at higher temperatures than those of VCO, with the first crystallization peak of RBD being markedly higher. In addition, the melting peaks of RBD consistently occurred at higher temperatures compared with VCO. These phenomena can be ascribed to the higher proportion of high-melting TAGs present in RBD, which is supported by the TAG profile shown in Fig. 1B and is consistent with the findings of Sonwai and Luangsasipong (2013). All physical treatments induced a shift to lower temperatures in melting peaks and corresponding melting point depression for both oils. The synergistic treatment caused the greatest depression, lowering the melting point from 25.25 °C (VCO-CK) to 23.98 °C (VCO-U-M) for VCO and from 26.86  °C (RBD-CK) to 24.64  °C (RBD-U-M) for RBD. This behavior was potentially related to the generation of small and uniform crystals, which leads to accelerated melting (Da Silva & Danthine, 2022). It may also be associated with the suppression of high-melting β polymorphs under mechanical stirring or ultrasonic treatment (West & Rousseau, 2020). Consistent with these mechanisms, DSC analysis revealed a lower melting point in the treated groups, which agrees with the XRD data indicating an increased proportion of low-melting-point β' polymorph.

### 3.6 Effects of different crystallization conditions on the textural properties

Hardness is a key texture attribute in semi-solid fat, which is directly related to consumer acceptance. Its value is determined by the composition of triacylglycerols, solid fat content, crystal morphology and polymorphism. Hardness, together with adhesion and cohesion, constitutes an important mechanical parameter of the lipid system (Nicholson et al., 2022). Deformation resistance, interface adhesion and crystal network stability determine the processing performance, taste and storage stability (Cui et al., 2022). Table 2 shows that the hardness increased (*p* < 0.05) after mechanical stirring and ultrasonic treatment, and the hardness of synergistic treatment was the highest. The hardness of the control sample was the lowest (VCO-CK was 16.62 N, RBD-CK was 19.9 N), while the hardness of the combined processed sample reached 35.40 N (VCO-UP-M) and 52.39 N (RBD-U-M). Both ultrasound and mechanical stirring can enhance the crystal hardness of coconut oil, and the synergistic effect is more significant. Ultrasound refines crystals, while mechanical stirring promotes crystallization uniformity through shear force. These tiny crystals enhance interfacial interactions and form a tight network, thereby significantly improving (*p* < 0.05) hardness (Suzuki et al., 2010). Under identical conditions, the hardness of RBD samples was consistently higher than that of VCO samples. This disparity is likely rooted in differences in lipid composition and crystal lattice integrity. As established in Section 3.1, the refining process removes indigenous minor components that act as interfacial impurities. In VCO, these molecules adsorb onto the crystal growth front and induce lattice defects, hindering orderly molecular packing. Conversely, the refined composition of RBD facilitates more contiguous and ordered triacylglycerol stacking, resulting in a 3D network with enhanced structural integrity and stronger inter-crystalline interactions.

**Table 2:** Texture parameters of VCO and RBD under different crystallization treatments.

Groups		Hardness (N)	Adhesiveness	Cohesiveness
VCO	CK	16.62 ± 0.92^gB^	−150.01 ± 35.10^dA^	0.18 ± 0.00^fA^
	M	22.61 ± 3.16^efA^	−66.38 ± 30.56^abcB^	0.24 ± 0.01^defA^
	UP	25.90 ± 0.12^deA^	−54.40 ± 2.01^abA^	0.23 ± 0.01^efB^
	U	30.69 ± 3.56^bcdA^	−26.00 ± 9.73^aA^	0.28 ± 0.03^bcdeA^
	UP-M	35.40 ± 0.17^bB^	−27.17 ± 19.86^abA^	0.32 ± 0.05^bcdA^
	U-M	33.53 ± 2.21^bcB^	−45.36 ± 1.24^abB^	0.35 ± 0.02^bA^
RBD	CK	19.90 ± 1.33^fgA^	−110.36 ± 45.54^bcdA^	0.23 ± 0.05^defA^
	M	27.00 ± 1.43^deA^	−159.64 ± 21.60^dA^	0.24 ± 0.00^cdefA^
	UP	28.27 ± 2.07^cdeA^	−139.66 ± 34.89^cdA^	0.26 ± 0.01^bcdefA^
	U	34.54 ± 0.46^bA^	−54.91 ± 15.69^abA^	0.26 ± 0.1.0^cdefA^
	UP-M	49.13 ± 1.88^aA^	−86.93 ± 24.20^abcdA^	0.33 ± 0.03^bcdA^
	U-M	52.39 ± 2.16^aA^	−100.58 ± 20.54^abcdA^	0.45 ± 0.06^aA^
Oil type (O)		**	**	**
Treatment (T)		**	**	**
Interaction (O × T)		**	**	*

Results are presented as means ± standard deviation (*n* = 3). Different lowercase letters indicate significant differences among treatments and different capital letters indicate significant differences between oil types (*p* < 0.05). *p < 0.05, **p < 0.01.

The impact of physical treatments on adhesiveness varied between the two oils. In VCO, the increased crystal density significantly reduced adhesiveness, whereas this effect was less pronounced in RBD. This discrepancy likely stems from the refining process, which removes indigenous minor components (e.g., monoacylglycerols and phospholipids) and yields a more ordered, less defect-prone crystal matrix in RBD (Chai et al., 2018a; Gordon & Rahman, 1991). Excessive adhesiveness may cause an undesirable sticky sensation in the mouth, whereas insufficient adhesiveness can result in oil migration and structural weakness. An appropriate level of adhesiveness, however, imparts favorable spreadability and morphological stability to the product (Subroto et al., 2024). Cohesiveness is closely related to the degree of packing density in the fat crystal network. A value close to 1 indicates the enhancement of structural integrity and internal binding strength (Liu et al., 2024). In this study, the control samples showed relatively low cohesion, which was mainly caused by irregular crystal arrangement and high heterogeneity. On the contrary, the synergistic treatment groups showed significantly enhanced cohesion, which is likely to be related to the β' polymorph and the generation of small and uniformly distributed crystals, which minimizes structural vacancies and strengthens the 3D crystal network (Da Silva et al., 2020). Such a highly concentrated structure is less prone to mechanical damage or structural collapse during storage and processing (Chen et al., 2025).

### 3.7 Influence mechanism of ultrasound-stirring synergistic effect on the coconut oil crystal network structure

**Fig. 7:**
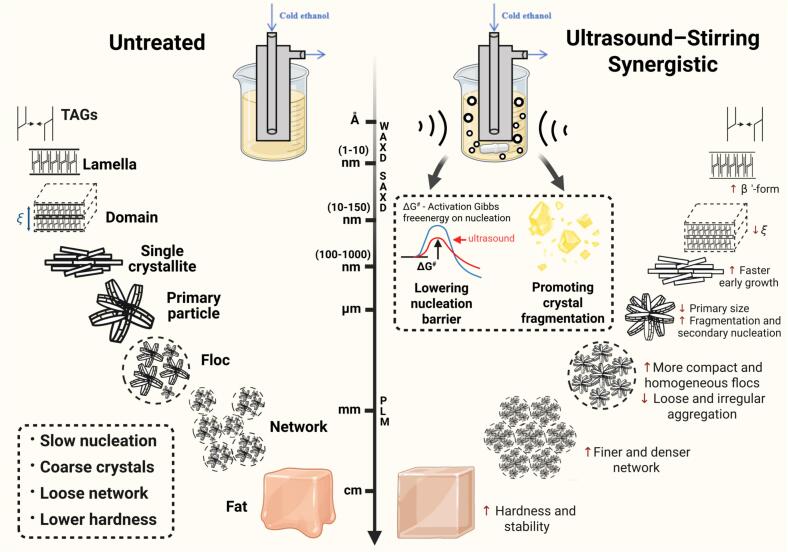
Schematic representation of the possible influence mechanism of ultrasound-stirring synergy on the coconut oil crystal network during the melting-crystallization process. WAXD: wide-angle X-ray diffraction; SAXD: small-angle X-ray diffraction; PLM: polarized light microscopy; ξ: crystallite thickness.

Coconut oil crystallization follows a hierarchical path: nucleation, layered structure formation, crystallite development and three-dimensional network assembly (Tran & Rousseau, 2016). The synergistic modulation of ultrasound and mechanical stirring promotes this process through the combination of cavitation-induced nucleation and enhanced mass transfer driven by shear force (Xiang et al., 2024). Fig. 7 is a schematic illustration of the proposed mechanism for ultrasound-stirring synergistic effect on coconut oil crystallization. The framework is proposed based on the structural evidence obtained in this study, integrating the cavitation-induced nucleation theory and the shear-enhanced mass transfer and fragmentation framework (Chai et al., 2018).

During early crystallization, ultrasonic cavitation reduced the nucleation energy barrier by generating high-energy nucleation centers (Gao & Meng, 2024), while mechanical stirring ensured a uniform spatial distribution of these nuclei, enabling high nucleation density and preventing localized clustering. This synergistic mechanism significantly shortens the induction time and accelerates initial crystal growth. As crystallization progressed, the combined physical field induced crystal fragmentation and rearrangement, thus slowing the later growth process. The process produced smaller and more uniform crystallites and narrowed the particle size distribution. Notably, this treatment prioritized the stable β' phase with low melting point, while nanoscale analysis showed that the crystallite thickness has been reduced. These structural optimizations eventually form a finer and more uniform crystal network, as evidenced by the high *D*_b_ value. Thermal analysis confirmed these structural changes. The DSC thermograms showed that the melting and crystallization peaks move to a lower temperature, consistent with the finer crystallites and enhanced molecular mobility. Fragmented crystals are reorganized into denser flocs at the nanoscale, where the inter-particle forces are stronger and the spatial packing efficiency is higher, forming a highly interconnected network. At the macro level, these changes endow the structured fats with superior hardness and structural integrity, primarily due to the denser packing of crystals and the enhanced binding forces between particles.

In summary, ultrasound-stirring synergy promotes the crystallization of coconut oil by simultaneously modulating nucleation, crystal growth, polymorphic selection and network assembly. The resulting structure is characterized by finer crystals, β’ phase dominance, reduced crystallite thickness, and a dense and mechanically stable fat crystal network.

## 4 Conclusion

This study showed that the synergistic application of ultrasound and mechanical stirring served as an effective physical method for regulating the crystallization process of coconut oil. In the early stage, synergistic treatment accelerated crystallization by improving the nucleation density and accelerating the initial crystal growth. In the later stage, shear-induced fragmentation further optimized the microstructure. Structural characterization showed that the ultrasound-stirring synergy promoted the formation of the β' polymorph, reduced the thickness of nanoscale crystallites, and produced finer and more uniform crystal aggregates exhibiting tighter spatial packing. These microstructural changes were accompanied by corresponding thermodynamic transitions, particularly a decrease in melting point and a shift in endothermic peaks. Among the tested protocols, the ultrasound-stirring approach delivered the most favorable outcomes, with the U-M treatment generating a well-interconnected crystal network that conferred optimal functional performance.

These findings demonstrate the potential of integrated physical fields as a sustainable and efficient alternative to chemical modification for tailoring the functionality of coconut oil. Practically, the enhanced hardness and improved structural stability of the resulting crystal network are highly suitable for the production of specialty fats, such as shortenings and margarines, where superior oil-binding capacity and smooth texture are essential. By modulating triacylglycerol assembly at molecular and meso-scales, this approach provides a basis for the precision design of clean-label, coconut oil-based fats. Such advancements are instrumental in developing functional fats with customized melting profiles and structural integrity, addressing the industry's demand for high-quality, sustainable alternatives to traditional hydrogenated fats. While this study provides a strong theoretical basis, further research is required to evaluate the scalability for industrial-scale production and the comparative effectiveness of this strategy against conventional tempering or seeding methods, as well as the long-term storage stability of the resulting crystal network.

## CRediT authorship contribution statement

**Huiting Yang:** Writing – original draft, Software, Methodology, Data curation. **Yuting Fu:** Methodology, Data curation. **Siyuan Xu:** Software, Investigation. **Jingya Xue:** Investigation, Formal analysis. **Jun Cao:** Writing – review & editing, Supervision.

## Declaration of competing interest

The authors declare that they have no known competing financial interests or personal relationships that could have appeared to influence the work reported in this paper.

## Data Availability

Data will be made available on request.
